# From Molecular Pathways to Artificial Intelligence: Advancing the Understanding and Management of Osteosarcopenia

**DOI:** 10.3390/ijms27177677

**Published:** 2026-08-27

**Authors:** Enrico Buccheri, Anastasia Xourafa, Martina Di Noto, Rita Chiaramonte, Antonino Catalano, Pietro Castellino, Michele Vecchio, Agostino Gaudio

**Affiliations:** 1 UOC Medicina Interna, Azienda Ospedaliera Universitaria Policlinico “G. Rodolico–San Marco”, 95123 Catania, Italy; enrico.buccheri@gmail.com; 2 UOSD Talassemia, Azienda Ospedaliera Universitaria Policlinico “G. Rodolico–San Marco”, 95123 Catania, Italy; axourafa@gmail.com; 3 UOC Medicina Generale, Presidio Ospedaliero “V. Emanuele”, 93012 Gela, Italy; mrtndinoto@gmail.com; 4 Department of Biomedical and Biotechnological Sciences, University of Catania, 95123 Catania, Italy; ritachiaramd@gmail.com (R.C.); michele.vecchio@unict.it (M.V.); 5 Department of Clinical and Experimental Medicine, University of Messina, 98124 Messina, Italy; catalanoa@unime.it; 6 Department of Clinical and Experimental Medicine, University of Catania, 95123 Catania, Italy; pcastell@unict.it

**Keywords:** osteosarcopenia, myokines, osteokines, artificial intelligence

## Abstract

Osteosarcopenia, defined as the coexistence of low bone mass and sarcopenia, is a multifactorial condition influenced by molecular crosstalk between bone and muscle. Growing evidence suggests that this interaction contributes to disease progression and highlights the need for integrated diagnostic and therapeutic approaches. Artificial intelligence (AI) is emerging as an important tool for the clinical management of osteosarcopenia, with potential applications in risk prediction, early diagnosis, evaluation of adverse outcomes such as fractures and falls, imaging analysis, and personalized treatment planning through machine learning and deep learning algorithms or with the support of large language models. This narrative review summarizes current evidence on the pathophysiological mechanisms underlying osteosarcopenia, with particular emphasis on the molecular mediators involved, and examines the current and potential applications of AI in its clinical assessment and management. By integrating advances in musculoskeletal biology with AI-driven approaches, this review highlights emerging opportunities to improve early detection, optimize clinical decision-making, and support the development of precision medicine strategies for patients with osteosarcopenia.

## 1. Introduction

The term osteosarcopenia [1] evolved from the earlier term “sarco-osteoporosis” [2] and was introduced more than 10 years ago to describe a condition characterized by the concomitant occurrence of osteoporosis and sarcopenia [1,2,3]. According to the World Health Organization, osteoporosis is a disease characterized by low bone mass and microarchitectural alteration of bone tissue, leading to increased bone fragility and susceptibility to fracture, most commonly at the vertebral level or the femoral neck [4]. Sarcopenia, by contrast, is a progressive disorder defined by the simultaneous loss of muscle mass and muscle strength, leading to reduced physical performance and an increased risk of falls [5,6]. Both conditions are very common among older adults, especially women [3,7] and individuals over 65 years of age [7], and have been associated with a higher risk of other health problems and increased mortality rates [5,6]. These two conditions appear to share numerous underlying biological and biochemical causes, and may, in fact, even result from the same processes [8].

The majority of epidemiological studies have considered the condition of “sarcopenia plus osteoporosis” or “sarcopenia plus osteopenia/osteoporosis” to define the prevalence of osteosarcopenia. According to recent data, the prevalence of osteosarcopenia in adults varies widely by region, with an estimated prevalence of 18.5% worldwide and around 10–20% in Western countries [7]. Furthermore, on a global scale, osteosarcopenia prevalence increases with age—rising from 17.8% in individuals under 80 years to 24.8% in those over 80 years—and also differs between men (15.3%) and women (19.4%) [7].

Considering that the global elderly population is projected to reach two billion by 2050, the absolute number of individuals with osteosarcopenia is expected to rise, with the condition thus emerging as a new global public health crisis with significant costs for national health services [3].

Because of the exponentially increased risk of comorbidities and mortality in osteosarcopenia compared with either disorder alone [9,10], an in-depth understanding of the triggering mechanisms of this disease is crucial to enable early identification and effective management [3].

Recently, artificial intelligence (AI) has emerged as an important support tool across virtually all fields of human knowledge, particularly in medicine [11]. The application of AI to a condition with a high socio-health impact such as osteosarcopenia could provide meaningful assistance in the prediction, diagnosis, and management of this condition in clinical practice. Several AI techniques, including machine learning (ML), deep learning (DL), and large language models (LLMs), can be employed in the assessment of osteoporosis [12,13,14] and sarcopenia [15,16,17].

This review aims to provide a comprehensive and consistent overview of the current state of the art in osteosarcopenia, focusing on both its molecular mechanisms and the role of AI. In particular, it focuses on the potential role of innovative AI-based techniques—such as ML, DL, and LLMs—in supporting early diagnosis, risk prediction, and treatment decision-making in patients with osteosarcopenia. Furthermore, this review discusses the current evidence, clinical implications, limitations, and future perspectives related to the integration of AI into the management of this condition.

Regarding methodology, although this work is a narrative review, a systematic approach was adopted. Relevant literature was identified through major databases, including PubMed, Scopus, the Cochrane Library, and Web of Science. The search strategy incorporated multiple keywords, such as “osteosarcopenia,” “osteosarcopenia and muscle,” “osteosarcopenia and bone,” “osteosarcopenia and artificial intelligence,” “osteosarcopenia and machine learning,” “osteosarcopenia and deep learning,” and “osteosarcopenia and large language models.” The inclusion criteria comprised peer-reviewed journal articles published in English, including clinical studies, preclinical studies, and review articles, without any restrictions with respect to publication date.

## 2. Osteoporosis and Sarcopenia: Two Faces of Musculoskeletal Aging

Osteoporosis and its premorbid condition, osteopenia, share several important similarities with sarcopenia and muscle mass loss (formerly termed pre-sarcopenia), both in diagnostic criteria and in underlying pathological risk factors.

From a diagnostic perspective, both osteoporosis and sarcopenia rely on quantitative thresholds (“cutoffs”) to define premorbid states and overt disease, and both conditions are characterized by defined severity classifications [4,5]. Osteoporosis is classically diagnosed using bone mineral density (BMD) assessed by dual-energy X-ray absorptiometry (DEXA) [4]. According to World Health Organization criteria, normal bone mass is defined by a T-score of ≥−1.0 standard deviation (SD), osteopenia by a T-score between −1.0 and −2.5 SD, and osteoporosis by a T-score of ≤−2.5 SD at the lumbar spine, femoral neck, or total hip [4]. Severe (or established) osteoporosis is diagnosed when a T-score of ≤−2.5 SD is accompanied by one or more fragility fractures, underscoring the progressive nature of skeletal fragility [4].

Similarly, sarcopenia is diagnosed using stepwise criteria that incorporate muscle mass, muscle strength, and physical performance. Early definitions emphasized low muscle mass alone, leading to the concept of “pre-sarcopenia” [18]. Current consensus statements, such as those from the European Working Group on Sarcopenia in Older People (EWGSOP2) [5,6], define probable sarcopenia by low muscle strength, confirmed sarcopenia by the presence of both low muscle strength and low muscle quantity, and severe sarcopenia when poor physical performance is also present. Commonly used cutoffs include appendicular lean mass adjusted for height squared of <7.0 kg/m^2^ in men and <5.5 kg/m^2^ in women, handgrip strength of <27 kg in men and <16 kg in women, and gait speed of ≤0.8 m/s [5,6].

The parallel between osteopenia and low muscle mass is particularly striking. In both conditions, an early quantitative decline in tissue mass precedes clinically evident adverse outcomes, such as fractures or disability. These premorbid states are often asymptomatic yet prognostically significant because they confer an increased risk of progression to overt disease and related complications [19]. Moreover, in both disorders, severity is defined not only by further reductions in tissue quantity but also by the emergence of functional impairment—fractures in osteoporosis and reduced physical performance in sarcopenia [4,5].

Beyond diagnostic similarities, osteoporosis and sarcopenia share a broad spectrum of risk factors. Aging is the predominant nonmodifiable determinant for both conditions, driving hormonal changes, chronic low-grade inflammation, mitochondrial dysfunction, and reduced regenerative capacity of bone and muscle tissues [20,21]. Sex-related factors also play a critical role: postmenopausal estrogen deficiency accelerates bone loss while simultaneously contributing to muscle atrophy, partially explaining the higher prevalence of both conditions in older women [4,5].

Lifestyle-related risk factors (i.e., physical inactivity), nutritional factors (i.e., vitamin D deficiency), and chronic diseases (i.e., chronic kidney disease, chronic obstructive pulmonary disease, diabetes mellitus, and inflammatory disorders) represent overlapping risk factors that promote both bone and muscle loss [4,5,8,22,23,24].

Frailty, defined both as a syndrome and as an accumulation of deficits or comorbidities, partially overlaps with sarcopenia through its association with declining muscle strength. Weight loss—a shared risk factor for osteoporosis and sarcopenia—is a core component, making frailty a critical outcome of advanced osteosarcopenia [25].

Long-term glucocorticoid therapy is a well-established cause of secondary osteoporosis and is also associated with accelerated muscle atrophy and weakness [26].

Moreover, both sarcopenia and osteoporosis share downstream clinical consequences, notably falls, fractures, loss of independence, and increased mortality [8]. The coexistence of low bone mass and reduced muscle function synergistically amplifies fracture risk, even in individuals with BMD values in the osteopenic range, highlighting the limitations of assessing bone health in isolation [8].

Osteogenic and myogenic activities are intricately regulated by a range of molecular mediators, including hormones (growth hormone, vitamin D, and sex steroids), myokines (such as irisin, myostatin, and activin), osteokines, and adipokines. Furthermore, these processes are influenced by nutritional factors, especially protein and carbohydrate intake, as well as calcium and vitamin D levels, and are profoundly affected by physical exercise [8].

Consequently, the pathophysiology of osteosarcopenia is fundamentally driven by disruptions in the physiological bidirectional communication, or crosstalk, between bone and muscle tissues [8]. It is well established that peak muscle mass and strength are typically achieved during early adulthood, coinciding with a peak in BMD [19,27]. This correlation reflects, in part, the biomechanical effects of muscle hypertrophy, whereby mechanical loading transmitted through muscle contractions stimulates bone formation, particularly at the epiphyseal level [28].

Similarly, during aging, reductions in muscle mass, muscle strength, and BMD follow parallel trajectories, underscoring their close interdependence [20]. In fact, the aging process itself, along with the chronic low-grade inflammatory state characteristic of later life—termed inflammaging—plays a crucial role in the pathogenesis of both osteoporosis and sarcopenia. This persistent inflammatory milieu disrupts normal bone–muscle homeostasis, ultimately fostering the development of osteosarcopenia [21].

Thus, osteoporosis and sarcopenia are two interconnected manifestations of musculoskeletal aging, sharing common pathogenic mechanisms such as chronic inflammation, hormonal dysregulation, and impaired bone–muscle crosstalk. This biological interdependence supports the concept of osteosarcopenia as a unified clinical entity rather than the simple coexistence of two age-related conditions. It is therefore conceivable that future risk prediction and diagnostic–therapeutic strategies could be integrated into a single algorithm, as proposed in the study by Kirk et al. [3].

## 3. Molecular Mechanisms of Paracrine and Endocrine Regulation: Myokines, Adipokines, Osteokines, and Endocrine Control

Muscle, bone, and adipose tissue communicate through tightly integrated bidirectional paracrine and endocrine signaling networks, which extend beyond simple mechanical coupling and constitute a functional musculoskeletal endocrine axis that coordinates tissue homeostasis, adaptation to mechanical loading, and energy metabolism [8]. Skeletal muscle is an active endocrine organ that releases myokines capable of modulating both bone remodeling and systemic metabolism [29]. Among these, interleukin-6 was one of the first myokines shown to increase after muscle contraction and to modulate bone turnover, particularly during physical exercise [30]. Similarly, irisin (derived from FNDC5), enhances osteoblast differentiation, regulates osteocyte sclerostin expression, reduces apoptosis, and simultaneously promotes muscle regeneration and adaptation to exercise, thereby representing a key mediator of muscle–bone communication [31,32,33]. Because of these pleiotropic effects, irisin has emerged as both a promising biomarker and a potential therapeutic drug for osteosarcopenia [31].

Growth and differentiation factors (GDFs), such as activin A and GDF8 (myostatin), are shared negative regulators of musculoskeletal homeostasis, limiting both muscle growth and bone formation [34,35]. Experimental inhibition of these ligands has been shown to induce muscle hypertrophy, reduce adiposity, enhance bone formation, and modify bone structure [34,35].

Several pharmacological strategies targeting the activin receptor pathway—including follistatin, ligand traps, and anti-receptor antibodies—have demonstrated anabolic effects on both muscle and bone in preclinical studies [36,37]. Ligand-trap approaches using soluble recombinant ActRIIA or ActRIIB have been shown to increase bone and muscle mass, as well as strength, in animal models, even under conditions of disuse atrophy [36,37]. These findings further support the concept that common molecular pathways simultaneously regulate both tissues.

An additional emerging mechanism of bone–muscle communication involves extracellular vesicles, particularly exosomes. These nanosized vesicles act as intercellular carriers of proteins, lipids, microRNAs, circular RNAs, and other bioactive molecules, thereby enabling the transfer of regulatory signals between bone, muscle, and other tissues [38].

In the context of osteoporosis, exosomes may influence osteoblast and osteoclast activity, angiogenesis, inflammation, and bone remodeling, suggesting a potential role in the regulation of skeletal homeostasis and disease progression [38]. Although their precise contribution to osteosarcopenia remains incompletely defined, exosomes may represent an important link between molecular bone–muscle crosstalk and the development of biomarkers.

In particular, molecular signatures derived from exosomes could potentially support early disease detection, risk stratification, and the identification of biologically distinct phenotypes, while engineered exosomes may be considered novel therapeutic strategies [38]. However, these applications remain investigational and require further validation in clinical studies.

Conversely, bone-derived osteokines also contribute to this bidirectional communication. Undercarboxylated osteocalcin improves muscle metabolism, exercise capacity, and interleukin-6 release during physical activity, whereas osteoglycin modulates muscle growth and glucose utilization [39]. Likewise, the RANK/RANKL/osteoprotegerin axis, long recognized as a central regulator of bone remodeling, also contributes to muscle integrity, and RANKL inhibition has been shown to alleviate muscle weakness and cachexia in experimental models [40,41].

Adipose tissue completes this regulatory network through the release of adipokines such as leptin and adiponectin, which integrate energy metabolism with chronic low-grade inflammation. These molecules influence both bone remodeling and muscle metabolism, highlighting the important role of adipose tissue in the pathophysiology of osteosarcopenia. Experimental findings indicate that adiponectin analogs may protect against bone and muscle loss during aging or in the context of estrogen deficiency [42].

At the systemic level, endocrine pathways reinforce this local crosstalk. Deficiencies in anabolic hormones, including sex steroids, growth hormone, insulin-like growth factor-1, and vitamin D, contribute to parallel declines in bone density and muscle strength [22]. Vitamin D regulates calcium absorption and directly affects muscle fibers through the vitamin D receptor, whereas growth hormone, insulin-like growth factor-1, and sex steroids collectively sustain musculoskeletal development, remodeling, and functional maintenance throughout life [23,43,44].

Overall, rather than acting through isolated pathways, myokines, osteokines, adipokines, and endocrine mediators form a highly interconnected signaling network in which alterations in one tissue rapidly influence the others. This integrated bone–muscle–adipose crosstalk provides a mechanistic explanation for the concurrent development of osteoporosis and sarcopenia during aging and identifies different molecular pathways that may represent future therapeutic targets for osteosarcopenia [8].

## 4. AI in Medicine: Definition, Components, and Clinical Implications

In recent years, AI has become a fundamental research support tool in the medical field, particularly in relation to disease prediction, early diagnosis, and estimation of therapeutic efficacy for complex age-related conditions such as osteosarcopenia, although it remains infrequently implemented in routine clinical practice [11,45]. Because osteosarcopenia results from the interaction of musculoskeletal, metabolic, and functional factors, it represents an ideal target for AI-based approaches capable of integrating heterogeneous clinical information into a single decision-support framework.

Unlike traditional rule-based methods, ML relies on data-driven model training rather than explicit programming. In different medical fields, including the context of osteosarcopenia, supervised ML algorithms can integrate clinical variables (i.e., BMD derived from DEXA, body composition parameters, muscle strength, physical performance tests, laboratory biomarkers, and imaging features) to identify individuals at high risk of disease, falls, fractures, and functional decline [45].

Advanced ML models, particularly DL algorithms based on artificial neural networks, are well suited to analyzing the complex and heterogeneous data generated in modern clinical practice—such as clinical notes, medical images, sensor data, and genomic information—to support clinically relevant predictions [45]. For osteosarcopenia, these multimodal approaches may improve automated image interpretation, quantify muscle quality and bone characteristics, and combine radiological findings with clinical and functional information to support earlier diagnosis and more accurate risk stratification.

Generative AI (GenAI) represents an evolution of ML systems, capable of producing new content by learning from large datasets, rather than merely performing classification or prediction tasks. LLMs are a key example of generative AI, consisting of models trained on large text corpora to generate coherent and contextually appropriate language through probabilistic modeling and alignment techniques [46]. Within osteosarcopenia, for example, these technologies could assist clinicians by integrating clinical guidelines with patient characteristics, generating personalized exercise and nutritional recommendations, summarizing longitudinal clinical data and supporting the preparation of patient education materials to improve adherence to long-term treatment programs.

At the clinical level, the integration of ML, DL, and LLMs into practice carries substantial implications for healthcare delivery, particularly in enhancing diagnostic accuracy, workflow efficiency, and clinical decision-making processes [11]. In osteosarcopenia, AI has the potential to integrate imaging, biochemical, functional, and clinical data into comprehensive predictive models capable of supporting early diagnosis, estimating fracture and fall risk, predicting treatment response, and monitoring disease progression over time. However, the clinical use of these technologies remains limited by issues related to generalizability, data quality, and integration into real-world settings, as well as concerns regarding transparency, bias, and accountability. Consequently, while these AI approaches have the potential to improve clinician performance and patient outcomes, their implementation requires rigorous validation, robust regulatory frameworks, and careful consideration of ethical and human-centered factors [11].

A key future direction for osteosarcopenia research could therefore be the development of biologically informed, multimodal AI models that integrate complementary domains rather than treating molecular, imaging, functional, and clinical information as independent data streams [45]. Molecular markers reflecting bone–muscle crosstalk and systemic inflammation, could provide information on the biological mechanisms underlying individual disease trajectories, whereas imaging-derived features could quantify BMD, muscle mass, and muscle quality [8,11]. These data could be integrated with functional measures, such as handgrip strength and gait speed, together with clinical risk factors, comorbidities, nutritional status, history of falls or fragility fractures, and validated frailty measures [8,11].

In this framework, AI would not merely reproduce conventional diagnostic thresholds but could identify multidimensional phenotypes that capture the biological and functional heterogeneity of osteosarcopenia. For example, a model could combine low BMD and reduced muscle quantity with specific molecular signatures, impaired physical performance, and frailty to distinguish patients with different probabilities of falls, fractures, disability, or mortality. Importantly, the same multimodal architecture could be extended longitudinally by incorporating treatment-response data, including changes in muscle strength, physical performance, BMD, body composition, biomarker profiles, adherence, and adverse events [45,46].

This approach would provide a direct mechanistic link between the molecular biology of bone–muscle crosstalk and computational prediction, with the potential to transform osteosarcopenia management from the parallel treatment of osteoporosis and sarcopenia into an integrated precision-medicine framework.

**Figure 1:**
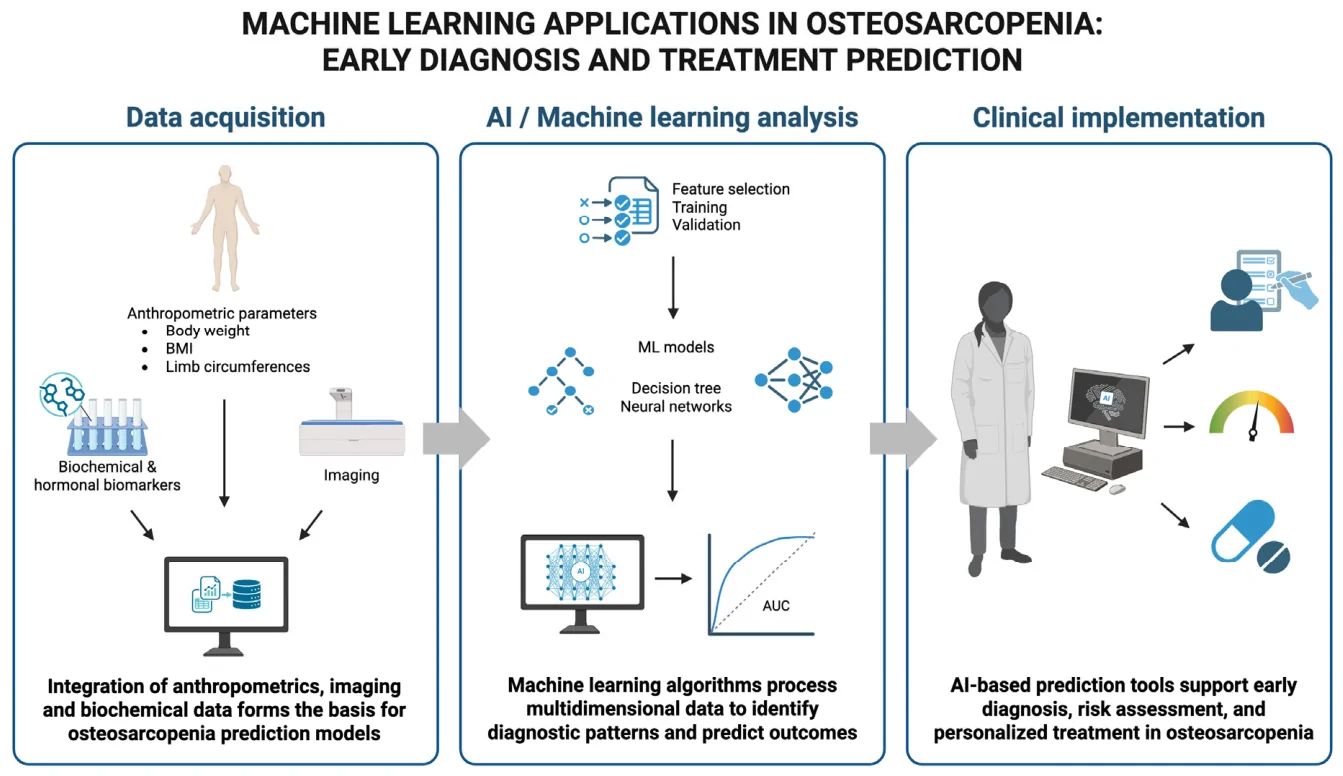
Schematic overview of the ML workflow for the early diagnosis and treatment prediction of osteosarcopenia. The figure illustrates the three main stages of the proposed framework: (i) **data acquisition**, integrating anthropometric parameters (e.g., body weight, body mass index, and limb circumferences), biochemical and hormonal biomarkers, and imaging data; (ii) **AI/ML analysis**, including feature selection, model training and validation, and the application of ML algorithms (e.g., decision trees and neural networks) to identify multidimensional diagnostic patterns and predict clinical outcomes; and (iii) **clinical implementation**, where AI-based prediction tools support early diagnosis, risk stratification, prognosis assessment, and personalized treatment decision-making for patients with osteosarcopenia. Performance is evaluated using standard metrics such as the area under the receiver operating characteristic curve. Created in BioRender. Gaudio, A. (2026) https://BioRender.com/cbr196o.

Ultimately, the future of AI in osteosarcopenia lies in the development of integrated multimodal platforms that combine imaging, body composition, muscle function, laboratory biomarkers, and electronic health records, into a unified clinical decision-support system. Such models could support opportunistic screening, facilitate earlier diagnosis, personalize exercise, nutritional and pharmacological interventions, and continuously monitor treatment response, thereby enabling precision management of osteosarcopenia (Figure 1).

## 5. ML Applications in Osteosarcopenia: Screening, Early Diagnosis and Treatment Prediction

In the context of osteoporosis and sarcopenia, several ML models have been proposed and validated with the aim of predicting the presence of these diseases [12,15,47,48] and forecasting treatment outcomes [49,50].

For example, assessment of muscle mass is highly challenging in clinical practice. Therefore, the ability to perform early screening of individuals with low muscle mass without the use of DEXA could represent an important strategy for initiating treatment as early as possible [5]. Several ML-based tools have been developed to address this issue, highlighting the importance of anthropometric variables such as body weight, body mass index, and limb circumference (thighs, arms, and calves) as key predictors for estimating muscle mass [51,52,53].

For instance, in a 2023 study [48] employing a specific ML approach based on decision tree algorithms, and in a subsequent 2024 study [15] that utilized ML software called the “Brain Project” [54,55]—which integrates evolutionary algorithms with neural networks—ML-based tools achieved accuracy levels of approximately 90% in terms of area under the curve (AUC) compared with whole-body DEXA measurements. Moreover, the 2024 study [15] showed that even a simple equation based solely on patient body weight could achieve high accuracy relative to DEXA, ranging from 85% to 89%, depending on sex and whether the study population consisted of the general or elderly population. Such ML-based methods could therefore represent extremely simple yet powerful tools to support clinical practice, enabling the early diagnosis of sarcopenia and providing useful assistance in monitoring the effects of rehabilitative or therapeutic interventions [15].

ML is also emerging as an increasingly important tool in the diagnosis of osteoporosis, as highlighted by a recent 2025 review [56]. ML-based approaches may be effectively employed for disease screening, as demonstrated by Bui et al. [57], who identified clinical variables such as body weight, height, and age as important contributors to a predictive algorithm that achieved an AUC of 0.81.

Moreover, similar ML models have been developed to assess the risk of developing sarcopenia and osteoporosis [58,59], as well as to predict and evaluate the efficacy of osteoporosis and sarcopenia treatments [49,50].

Although ML appears capable of substantially supporting clinicians in diagnostic and therapeutic decision-making, several issues remain unresolved, including the “black-box” nature of many ML models and the need for proper validation across different population cohorts with adequate sample sizes [45,56].

Beyond the encouraging results reported by individual studies, the current body of evidence requires careful critical interpretation before ML models can be translated into routine clinical practice.

First, model performance varies considerably according to the target outcome, input variables, study population, and validation strategy, making direct comparisons difficult. While several algorithms have reported good discriminative ability, with AUC values generally ranging from approximately 0.80 to 0.90 for screening purposes, these results are frequently obtained in retrospective single-center cohorts or within the same population used for model development [15,48,57]. Consequently, the risk of overfitting and optimistic performance estimates cannot be excluded.

Moreover, models based on simple anthropometric variables offer the important advantage of being inexpensive, easily applicable, and suitable for large-scale screening, although they may sacrifice some biological information compared with multimodal approaches integrating imaging, biochemical, and functional data [45]. Conversely, more complex ML models may achieve higher predictive performance but often suffer from limited interpretability, greater computational requirements, and reduced feasibility for implementation in primary care settings [45].

Another important limitation concerns external validation. Only a limited number of studies have evaluated their algorithms in independent cohorts with different demographic characteristics, healthcare systems, or ethnic backgrounds. This is particularly relevant for osteosarcopenia, whose prevalence, body composition, and clinical presentation vary substantially across populations [3]. Consequently, models developed in specific geographic or clinical settings may not maintain the same diagnostic performance when applied elsewhere.

Future studies should therefore prioritize large multicenter prospective investigations, standardized reporting of model development and validation, calibration analyses, and comparisons with established clinical prediction tools. Addressing these methodological and translational challenges will be essential before ML-based approaches can be safely incorporated into the routine management of osteosarcopenia.

## 6. DL: Image Analysis, Technical Performance, and Clinical Implications

DL, particularly convolutional neural networks, has shown considerable potential for the automated analysis of medical images relevant to sarcopenia and osteoporosis [45]. However, the interpretation of DL performance requires a clear distinction between technical image-analysis endpoints and clinically meaningful outcomes. In particular, segmentation accuracy, diagnostic accuracy, risk prediction, treatment-response prediction, and improvement in patient outcomes represent different levels of evidence and should not be considered interchangeable.

In the context of sarcopenia, conventional neural network-based architectures, including U-Net-derived models, have been used to segment skeletal muscle on routinely acquired computer tomography (CT) examinations and to quantify muscle area and quality. Reported Dice similarity coefficients frequently exceed 0.94–0.95, indicating a high degree of spatial overlap between automated and reference segmentations [16]. These results demonstrate strong technical performance in identifying anatomical structures and extracting quantitative imaging features. However, a high Dice coefficient does not, by itself, establish that the model improves the diagnosis of sarcopenia, predicts fractures or falls, influences treatment selection, or improves patient outcomes. Accordingly, the clinical relevance of automated segmentation depends on the subsequent validity of the derived measurements and their association with clinically meaningful endpoints.

A similar distinction applies to opportunistic CT-based assessment of osteoporosis. DL models can automatically identify vertebral structures and quantify trabecular attenuation, potentially enabling the extraction of bone-related information from CT examinations performed for other clinical indications [13]. These studies primarily demonstrate the technical feasibility and measurement performance of automated image analysis. Diagnostic usefulness requires additional evidence, including comparison with an accepted reference standard such as DEXA, evaluation of sensitivity and specificity or other discrimination measures, calibration, and validation in clinically representative populations. Moreover, the generalizability of CT-based models may be affected by differences in acquisition protocols, scanner manufacturers, reconstruction algorithms, and patient characteristics [13].

The next level of evidence concerns prognostic and risk-prediction performance. Imaging-derived measures such as skeletal muscle index, muscle attenuation, and vertebral trabecular density may be associated with clinically relevant outcomes, including postoperative complications, functional decline, mortality, and fracture risk [13,16]. However, these associations should be distinguished from the performance of the segmentation algorithm itself. A model may accurately segment muscle or vertebral structures without necessarily providing clinically useful prediction of future events. Conversely, a prognostic model requires independent evaluation of discrimination, calibration, clinical utility, and, ideally, incremental value over established clinical predictors.

Generally, the interpretation of AI-based imaging studies should therefore consider the study population and sample size, the imaging modality and acquisition protocol, the input variables and reference standard, the specific algorithm and architecture, the training and testing strategy, and the method used to prevent data leakage and overfitting. External validation in geographically, demographically, and technically distinct populations is particularly important before clinical implementation. In addition, the distinction between retrospective technical validation and prospective evaluation in real-world clinical workflows should be made explicit [11,45].

## 7. LLMs in Osteosarcopenia: Patient Education, Communication, and Clinician Support

The evidence regarding the use of LLMs in sarcopenia and osteoporosis remains preliminary and should be interpreted according to the specific methodology of each evaluation. Recent comparative studies have assessed models such as ChatGPT, Gemini, and DeepSeek by submitting sets of disease-related questions concerning definitions, risk factors, diagnosis, prevention, and treatment and by evaluating the generated responses according to predefined criteria of correctness, completeness, and clinical relevance [14,17]. However, the interpretation of these findings requires careful consideration of the model version, the number and clinical domain of the questions, the prompt formulation, the reference answers or guidelines used as the benchmark, and the method adopted for expert scoring. Differences in these methodological factors may substantially influence the reported performance and limit direct comparisons between studies.

In the context of sarcopenia, available evidence suggests that LLMs may provide useful information for patient-oriented questions and may facilitate the communication of complex concepts related to muscle health, physical activity, nutrition, and prevention. Nevertheless, the performance of an LLM in answering a predefined set of questions should not be equated with diagnostic accuracy or clinical effectiveness. In particular, a correct answer to a general educational question does not demonstrate the ability to diagnose sarcopenia, determine disease severity, select an individualized exercise or nutritional intervention, or predict treatment response. These applications require the integration of patient-specific information, validated diagnostic criteria, clinical examination, functional assessment, and professional clinical judgment [17].

Similarly, comparative evaluations of LLMs in osteoporosis have reported generally favorable performance in responding to patient-oriented questions, with assessments based on dimensions such as accuracy and content completeness [14]. However, the clinical meaning of these results remains limited when the evaluation is based exclusively on static question–answer datasets. Such studies do not necessarily establish how the models perform in real-world clinical interactions, how consistently they respond to repeated or differently phrased prompts, or whether their outputs improve patient understanding, adherence, diagnostic accuracy, or treatment outcomes. Accordingly, model version, prompt design, number and type of questions, scoring methodology, expert agreement, and the criteria used to define accuracy should be explicitly reported in future studies to improve reproducibility and facilitate meaningful comparisons between LLMs [14,17].

A further concern is the possibility of hallucinations, defined as the generation of plausible but inaccurate, unsupported, or fabricated information. In the context of osteosarcopenia, hallucinated recommendations may be particularly problematic because patients may interpret apparently confident responses as personalized medical advice. Potential risks include incorrect interpretation of diagnostic criteria, inappropriate exercise or nutritional recommendations, inaccurate information regarding pharmacological treatments, and failure to recognize situations requiring medical assessment. These risks are amplified by the heterogeneous clinical presentation of older adults, the frequent presence of multimorbidity and polypharmacy, and the potential vulnerability of patients with frailty or cognitive impairment. Therefore, LLM outputs should be regarded as probabilistic informational content rather than as independently validated clinical recommendations [14,17].

The most defensible current role of LLMs in osteosarcopenia is therefore as a complementary support tool for patient education, health communication, literature searching, guideline-based information retrieval, translation and simplification of medical information, and clinician drafting of educational or clinical materials. LLMs may also assist healthcare professionals in organizing information and generating preliminary summaries, provided that all outputs are critically reviewed and verified against authoritative sources. Their use should remain embedded within a human-supervised, evidence-based framework in which final responsibility for diagnosis and treatment decisions remains with qualified healthcare professionals [14,17].

A recent review [60] highlights that GenAI-based systems, particularly multimodal conversational agents integrating text, voice, images, and sensor-derived data, have the potential to support personalized health management, promote adherence to lifestyle interventions, facilitate remote monitoring, and improve patient engagement. These capabilities are particularly applicable to sarcopenia, where long-term management relies on sustained adherence to individualized exercise programs, nutritional optimization, and continuous follow-up. By delivering tailored recommendations, providing motivational support, and integrating data from wearable devices and remote monitoring technologies, GenAI could enhance self-management while reducing the burden on healthcare systems [60].

Nevertheless, the review [60] also emphasizes that current evidence remains limited, with most studies involving cognitively healthy older adults and highlighting persistent challenges related to validation, usability, ethical considerations, data privacy, and algorithmic bias. Overall, this review supports the emerging concept that generative AI may become a valuable component of integrated geriatric care, offering scalable and personalized interventions that could also improve the prevention and management of sarcopenia. However, disease-specific clinical studies are still needed to establish its effectiveness and safety in this setting [60].

Future studies should move beyond isolated accuracy scores and evaluate LLMs using standardized, transparent, and clinically relevant methodologies. These studies should report the exact model version, prompting strategy, question number and content, reference standards, expert assessment procedures, interrater agreement, accuracy and completeness criteria, and the frequency and severity of hallucinations. Importantly, evaluation should also include patient-safety outcomes, usability, health-literacy considerations, and prospective testing in real-world clinical or patient-education settings.

## 8. Clinical Management: Current Evidence and Future Perspectives

To date, no pharmacological therapies have been specifically approved for osteosarcopenia. Thus, in current clinical practice, osteosarcopenia is essentially managed as two distinct conditions—sarcopenia and osteoporosis—although with several overlapping therapeutic aspects [3].

Because of this, a diagnostic–therapeutic algorithm has been proposed to enable the simultaneous assessment and management of both conditions [3].

From a practical clinical perspective, the management of osteosarcopenia should begin with the early identification of individuals at risk through opportunistic case finding in older adults, particularly those with a history of fragility fractures, recurrent falls, frailty, or functional decline [3,4,5]. A stepwise diagnostic pathway is recommended, combining the assessment of muscle strength (e.g., handgrip strength or chair stand test), physical performance (e.g., gait speed or Short Physical Performance Battery), and muscle quantity or quality using validated imaging techniques, together with BMD assessment by DEXA and fracture risk evaluation [3,4,5]. This integrated diagnostic approach allows clinicians to characterize both the skeletal and muscular components of the disease and to tailor treatment according to disease severity and individual patient characteristics [3].

For both conditions, therapeutic exercise plays a pivotal role, representing a curative intervention for sarcopenia and an essential adjunctive therapy for osteoporosis [4,5,61,62]. Therapeutic exercise is considered both the first-line treatment and a fundamental preventive strategy for osteosarcopenia [3,8]. Multiple systematic reviews have confirmed the beneficial effects of therapeutic exercise in patients with osteosarcopenia, showing that the combination of aerobic, resistance, and balance training improves muscle mass and strength, BMD, and quality of life, while reducing fall risk and providing pain relief [61,62].

In addition to exercise, vitamin D supplementation plays an important complementary role in the management of both conditions [63,64]. In particular, the efficacy of vitamin D supplementation in osteoporosis, with or without concurrent calcium supplementation, has been well established [64]. Vitamin D has been shown to promote proper bone mineralization and optimal calcium utilization, while preventing secondary hyperparathyroidism [8]. In the context of sarcopenia, vitamin D is thought to support a microenvironment favorable for muscle activation [63]. Thus, supplementation with approximately 1000 IU/day of vitamin D is currently recommended, although specific evidence supporting this dosage remains limited [3,5].

Calcium supplementation is also considered important because calcium is essential for both adequate bone mineralization and muscle contraction [65]; an intake of 500–600 mg/day is generally recommended [3].

Furthermore, combining therapeutic exercise with protein–amino acid supplementation has shown efficacy in improving osteosarcopenia outcomes, particularly with leucine at >1.2 g/day [66] and creatine (3–5 g/day) [67], both of which are key substrates involved in muscle tissue synthesis. Such supplementation is therefore recommended as an adjunct to therapeutic exercise in patients with sarcopenia and, consequently, osteosarcopenia [3].

Given the multifactorial nature of osteosarcopenia, optimal management requires a multidisciplinary approach involving internists, geriatricians, endocrinologists, physiatrists, physiotherapists, nutritionists, primary care physicians, and, when appropriate, orthopedic surgeons [68]. Multidisciplinary care facilitates the implementation of individualized exercise programs, optimization of nutritional status, management of osteoporosis-specific pharmacological therapies, prevention of falls, and treatment of comorbidities that may contribute to functional decline [68]. Patient education and strategies aimed at improving long-term adherence to exercise and nutritional interventions should also represent integral components of routine clinical care [68].

The therapeutic management of the osteoporotic component should follow the most recent clinical guidelines [4], combining therapeutic exercise—as previously emphasized—with pharmacological therapy, including antiresorptive or anabolic agents selected according to the patient’s BMD values, fracture risk, and fracture history.

Longitudinal monitoring is essential to evaluate treatment effectiveness and to adjust therapeutic strategies over time. Follow-up should include periodic reassessment of muscle strength, physical performance, nutritional status, fall occurrence, and BMD, with the frequency of evaluations tailored to the patient’s clinical condition and the therapeutic interventions implemented. Monitoring adherence to exercise, nutritional supplementation, and pharmacological treatment is equally important, because sustained adherence largely determines long-term clinical outcomes. This comprehensive follow-up strategy enables timely identification of disease progression or treatment failure and supports personalized therapeutic adjustments [3,4,5].

Recent studies have focused on potential pharmacological strategies designed to target the activin/myostatin signaling pathway because both activin and myostatin are recognized inhibitors of osteogenesis and muscle hypertrophy [36,37]. Promising preclinical results have been reported over recent years, including studies evaluating these inhibitors in combination with androgenic therapies [36,37]. Moreover, preliminary findings from a phase I clinical trial demonstrated that combined blockade of GDF8 and activin with specific antibodies induced greater muscle growth than either antibody alone, together with a concomitant reduction in fat mass [69]. These results may pave the way for the development of new therapeutic options for osteosarcopenia.

Finally, findings from several preclinical studies suggest that in addition to its potential role as a biomarker for sarcopenia, irisin may also serve as a therapeutic agent for the treatment of osteosarcopenia. Although clinical data are not yet available, irisin is a potent stimulator of both osteogenesis and muscle hypertrophy and therefore represents a promising pharmacological candidate [31,70,71,72,73].

Beyond pharmacological and biologically targeted therapies, biomaterial-based strategies represent another emerging direction for musculoskeletal repair and regeneration [74]. Osteosarcopenia should not be viewed solely as a diagnostic or risk-prediction problem, but also as a condition characterized by impaired tissue maintenance, repair, and regenerative capacity.

Advanced biomaterials may provide multifunctional platforms capable of supporting tissue regeneration while simultaneously addressing complications that can compromise healing [74].

For example, Nguyen et al. [74] developed hydroxyapatite scaffolds coated with silver–gallium liquid-metal nanoparticles that combined antimicrobial activity with bone-regenerative potential. The system showed activity against clinically relevant pathogens, through mechanisms involving oxidative stress, bacterial membrane damage, ATP depletion, and disruption of essential bacterial processes [74]. In vivo, the biomaterial reduced bacterial colonization and improved tissue integration, highlighting the potential of multifunctional scaffolds to simultaneously address infection-related complications and impaired bone regeneration [74].

Although this study was not specifically conducted in patients with osteosarcopenia, it illustrates how advanced biomaterials could eventually contribute to integrated strategies for musculoskeletal repair in individuals with compromised bone and muscle homeostasis. Nevertheless, these approaches remain largely investigational in the context of osteosarcopenia.

Importantly, the available therapeutic strategies should be distinguished according to the strength and maturity of the supporting clinical evidence. Established clinical management of osteosarcopenia currently relies primarily on structured therapeutic exercise, fall-prevention strategies, adequate nutritional intake—including sufficient protein and, when appropriate, supplementation with vitamin D, calcium, leucine, or creatine—and osteoporosis-directed pharmacotherapy based on current clinical guidelines and individual fracture risk [3,4,5]. These interventions have the strongest clinical foundation and should therefore constitute the core of routine patient management [3]. Conversely, several approaches discussed in the context of osteosarcopenia remain investigational or should be considered future therapeutic perspectives. These include myostatin/activin pathway inhibition [69], irisin-based therapies [31,70,71,72,73], exosome-based interventions [38], biomaterial-assisted tissue regeneration [74], and AI-guided personalized treatment strategies [14,17].

Although these approaches are biologically promising and may eventually contribute to integrated precision management, their current clinical applicability remains limited by the lack of sufficient high-quality clinical evidence, including robust randomized trials, external validation, and demonstrated improvements in patient-centered outcomes. Accordingly, these emerging strategies should not yet be considered established treatments but rather promising avenues for future research and clinical translation.

In particular, in the context of sarcopenia and osteoporosis, AI could, in the future, serve as a tool for risk prediction, diagnostic support, and treatment outcome prediction through the appropriate integration of available data. However, important limitations remain, particularly regarding large-scale implementation in clinical practice, ethical considerations, and the quality of the data used [75,76].

**Figure 2:**
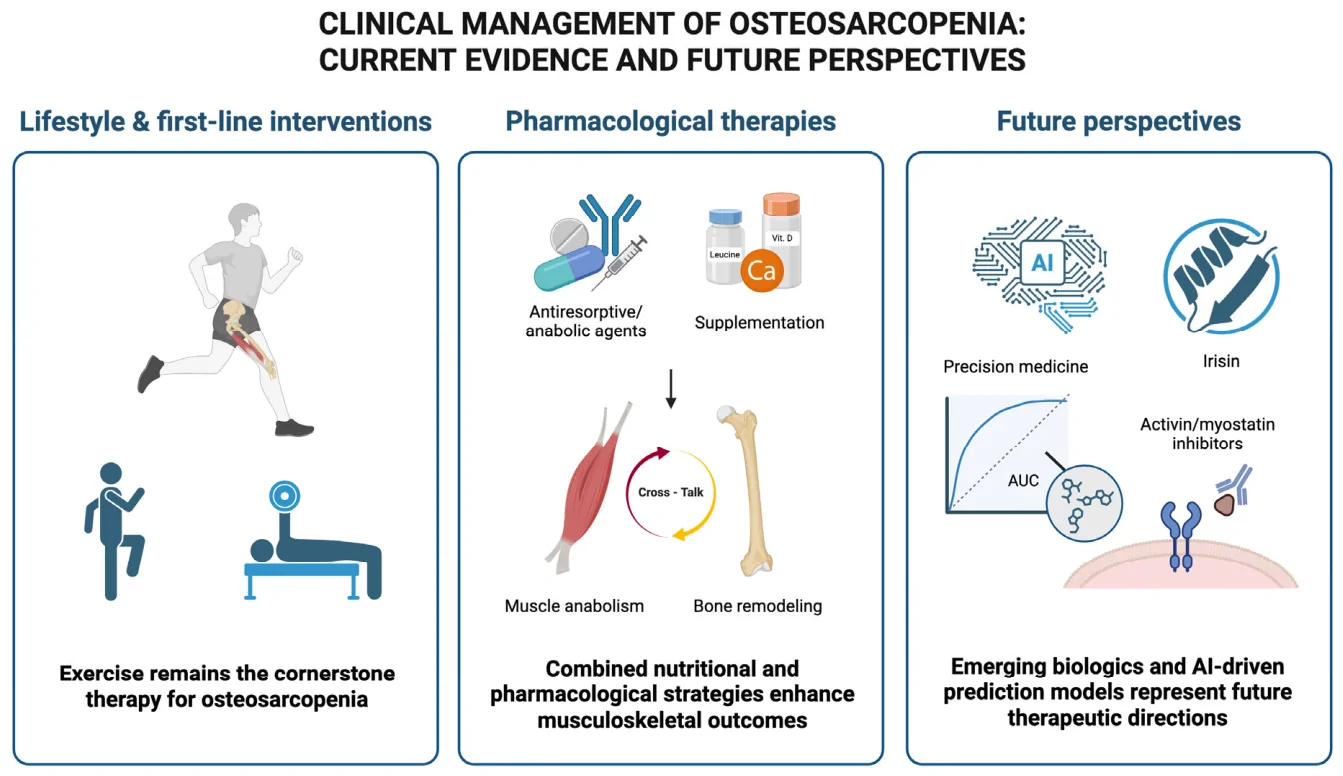
Overview of the current clinical management of osteosarcopenia and emerging therapeutic perspectives. The figure summarizes the principal components of a comprehensive management strategy for osteosarcopenia. **Lifestyle and first-line interventions** emphasize exercise, particularly resistance and multicomponent training, as the cornerstone of treatment to improve muscle strength, physical performance, and bone health. **Pharmacological therapies** include antiresorptive and anabolic agents, together with nutritional supplementation (e.g., calcium, vitamin D, and protein or amino acid supplementation), aimed at enhancing the functional crosstalk between muscle and bone, promoting muscle anabolism, and supporting bone remodeling. **Future perspectives** highlight the potential of precision medicine approaches, AI-based predictive models, and emerging biological targets, including irisin and activin/myostatin pathway inhibitors, to improve risk stratification, optimize therapeutic decision-making, and enable personalized management of osteosarcopenia. Predictive model performance is exemplified by the area under the receiver operating characteristic curve. Created in BioRender. Gaudio, A. (2026) https://BioRender.com/tlusnly.

Figure 2 summarizes current management strategies for osteosarcopenia, including physical exercise, pharmacological treatments, and nutritional interventions, and highlights future perspectives regarding integrated osteomuscular pharmacological approaches and the role of AI.

## 9. Conclusions

Osteosarcopenia is a prevalent and complex condition associated with a high risk of comorbidity and mortality. The shared molecular pathophysiology of osteoporosis and sarcopenia has enabled a deeper understanding of their common risk factors and potential therapeutic targets.

Current evidence indicates that AI represents a promising tool in the management of osteosarcopenia. AI-based approaches, including ML, DL, and LLMs, have shown potential in improving risk stratification, enabling earlier diagnosis through multimodal data integration, and supporting personalized treatment strategies. Moreover, these technologies may enhance patient education and clinical decision-making. However, current evidence remains limited and heterogeneous, with challenges related to validation, interpretability, and clinical integration. Further robust studies are therefore needed to confirm their effectiveness and ensure safe implementation.

In current clinical practice, physical exercise, nutritional supplementation, and targeted pharmacological treatment for osteoporosis constitute the main therapeutic strategies for osteosarcopenia. Future perspectives include the development of pharmacological agents capable of simultaneously promoting muscle hypertrophy and bone mineralization, with the aim of addressing both components of osteosarcopenia.

The main priorities for future research include prospective studies, clinical validation of AI tools, and evaluation of their impact on clinical outcomes, underscoring the importance of translating these advances into clinical practice.

## Data Availability

No new data were created or analyzed in this study. Data sharing is not applicable to this article.

## Abbreviations

The following abbreviations are used in this manuscript:

AI	Artificial intelligence
AUC	Area under the curve
BMD	Bone Mineral Density
CT	Computed Tomography
DEXA	Dual-energy X-ray absorptiometry
DL	Deep Learning
EWGSOP2	European Working Group on Sarcopenia in Older People
GDFs	Growth and differentiation factors
GenAI	Generative artificial intelligence
LLMs	Large language models
ML	Machine Learning

## References

[1] Huo Y.R., Suriyaarachchi P., Gomez F., Curcio C.L., Boersma D., Muir S.W., Montero-Odasso M., Gunawardene P., Demontiero O., Duque G. (2015). Phenotype of Osteosarcopenia in Older Individuals with a History of Falling. J. Am. Med. Dir. Assoc..

[2] Binkley N., Buehring B. (2009). Beyond FRAX^®^: It’s Time to Consider “Sarco-Osteopenia”. J. Clin. Densitom..

[3] Kirk B., Zanker J., Duque G. (2020). Osteosarcopenia: Epidemiology, Diagnosis, and Treatment—Facts and Numbers. J. Cachexia Sarcopenia Muscle.

[4] Morin S.N., Leslie W.D., Schousboe J.T. (2025). Osteoporosis. JAMA.

[5] Cruz-Jentoft A.J., Sayer A.A. (2019). Sarcopenia. Lancet.

[6] Cruz-Jentoft A.J., Bahat G., Bauer J., Boirie Y., Bruyère O., Cederholm T., Cooper C., Landi F., Rolland Y., Sayer A.A. (2019). Sarcopenia: Revised European Consensus on Definition and Diagnosis. Age Ageing.

[7] Chen S., Xu X., Gong H., Chen R., Guan L., Yan X., Zhou L., Yang Y., Wang J., Zhou J. (2024). Global Epidemiological Features and Impact of Osteosarcopenia: A Comprehensive Meta-Analysis and Systematic Review. J. Cachexia Sarcopenia Muscle.

[8] Gielen E., Dupont J., Dejaeger M., Laurent M.R. (2023). Sarcopenia, Osteoporosis and Frailty. Metabolism.

[9] Yoo J.I., Kim H., Ha Y.C., Kwon H.B., Koo K.H. (2018). Osteosarcopenia in Patients with Hip Fracture Is Related with High Mortality. J. Korean Med. Sci..

[10] Veronese N., Ragusa F.S., Sabico S., Dominguez L.J., Barbagallo M., Duque G., Al-Daghri N. (2024). Osteosarcopenia Increases the Risk of Mortality: A Systematic Review and Meta-Analysis of Prospective Observational Studies. Aging Clin. Exp. Res..

[11] Rajpurkar P., Chen E., Banerjee O., Topol E.J. (2022). AI in Health and Medicine. Nat. Med..

[12] Ullah K.A., Rehman F., Anwar M., Faheem M., Riaz N. (2023). Machine Learning-Based Prediction of Osteoporosis in Postmenopausal Women with Clinical Examined Features: A Quantitative Clinical Study. Health Sci. Rep..

[13] Westerhoff M., Gyftopoulos S., Dane B., Vega E., Murdock D., Lindow N., Herter F., Bousabarah K., Recht M.P., Bredella M.A. (2025). Deep Learning-Based Opportunistic CT Osteoporosis Screening and the Establishment of Normative Values. Radiology.

[14] Li X., Li G., Zhao Y., Liang Y., Dong Y., Zhang J. (2025). Exploring and Comparing the Use of Large Language Models in Supporting Osteoporosis Health Consultations. Clin. Interv. Aging.

[15] Buccheri E., Dell’Aquila D., Russo M., Chiaramonte R., Vecchio M. (2024). Appendicular Skeletal Muscle Mass in Older Adults Can Be Estimated with a Simple Equation Using a Few Zero-Cost Variables. J. Geriatr. Phys. Ther..

[16] Pei T., Lei Y., Gao Y., Zhang M., Xu T., Yang W., Wen Q., Liu Q. (2026). Artificial Intelligence-Driven Assessment of Sarcopenia in Orthopedic Geriatrics: Technical Progress and Clinical Implications. Front. Endocrinol..

[17] Huang T., Kirk B., Close J., Lim J.-Y., Duque G., Ebeling P., Yang M., Tian M., Chui C.S., Liu C. (2026). Evaluating the Performance of Large Language Models in Sarcopenia-Related Patient Queries: A Foundational Assessment for Patient-Centered Validation. Front. Aging.

[18] Cruz-Jentoft A.J., Baeyens J.P., Bauer J.M., Boirie Y., Cederholm T., Landi F., Martin F.C., Michel J.-P., Rolland Y., Schneider S.M. (2010). Sarcopenia: European Consensus on Definition and Diagnosis: Report of the European Working Group on Sarcopenia in Older People. Age Ageing.

[19] Sutter T., Toumi H., Valery A., El Hage R., Pinti A., Lespessailles E. (2019). Relationships between Muscle Mass, Strength and Regional Bone Mineral Density in Young Men. PLoS ONE.

[20] Qin H., Jiao W. (2022). Correlation of Muscle Mass and Bone Mineral Density in the NHANES US General Population, 2017–2018. Medicine.

[21] Nusrat S., Din R.U., Tariq M.A., Yang H. (2025). Epigenetic Dysregulation and Osteocyte Senescence: Convergent Drivers of Osteosarcopenia in Aging Bone and Muscle. Aging Dis..

[22] Laurent M.R., Dubois V., Claessens F., Verschueren S.M.P., Vanderschueren D., Gielen E., Jardí F. (2016). Muscle-Bone Interactions: From Experimental Models to the Clinic? A Critical Update. Mol. Cell. Endocrinol..

[23] Nakamura S., Sato Y., Kobayashi T., Kaneko Y., Ito E., Soma T., Okada H., Miyamoto K., Oya A., Matsumoto M. (2020). Vitamin D Protects against Immobilization-Induced Muscle Atrophy via Neural Crest-Derived Cells in Mice. Sci. Rep..

[24] Eller-Vainicher C., Cairoli E., Grassi G., Grassi F., Catalano A., Merlotti D., Falchetti A., Gaudio A., Chiodini I., Gennari L. (2020). Pathophysiology and Management of Type 2 Diabetes Mellitus Bone Fragility. J. Diabetes Res..

[25] Kim D.H., Rockwood K. (2024). Frailty in Older Adults. N. Engl. J. Med..

[26] LeBoff M.S., Greenspan S.L., Insogna K.L., Lewiecki E.M., Saag K.G., Singer A.J., Siris E.S. (2022). The Clinician’s Guide to Prevention and Treatment of Osteoporosis. Osteoporos. Int..

[27] Nguyen H.G., Pham M.T.D., Ho-Pham L.T., Nguyen T.V. (2020). Lean Mass and Peak Bone Mineral Density. Osteoporos. Sarcopenia.

[28] Moon R.J., Cole Z.A., Crozier S.R., Curtis E.M., Davies J.H., Gregson C.L., Robinson S.M., Dennison E.M., Godfrey K.M., Inskip H.M. (2015). Longitudinal Changes in Lean Mass Predict PQCT Measures of Tibial Geometry and Mineralisation at 6–7 Years. Bone.

[29] Florin A., Lambert C., Sanchez C., Zappia J., Durieux N., Tieppo A.M., Mobasheri A., Henrotin Y. (2020). The Secretome of Skeletal Muscle Cells: A Systematic Review. Osteoarthr. Cartil. Open.

[30] Pedersen B.K., Steensberg A., Fischer C., Keller C., Keller P., Plomgaard P., Febbraio M., Saltin B. (2003). Searching for the Exercise Factor: Is IL-6 a Candidate?. J. Muscle Res. Cell Motil..

[31] Paoletti I., Coccurello R. (2024). Irisin: A Multifaceted Hormone Bridging Exercise and Disease Pathophysiology. Int. J. Mol. Sci..

[32] Gaudio A., Rapisarda R., Xourafa A., Zanoli L., Manfrè V., Catalano A., Signorelli S.S., Castellino P. (2021). Effects of Competitive Physical Activity on Serum Irisin Levels and Bone Turnover Markers. J. Endocrinol. Investig..

[33] Yano N., Zhao Y.T., Zhao T.C. (2021). The Physiological Role of Irisin in the Regulation of Muscle Glucose Homeostasis. Endocrines.

[34] Chan A.S.M., McGregor N.E., Poulton I.J., Hardee J.P., Cho E.H.-J., Martin T.J., Gregorevic P., Sims N.A., Lynch G.S. (2021). Bone Geometry Is Altered by Follistatin-Induced Muscle Growth in Young Adult Male Mice. JBMR Plus.

[35] Latres E., Mastaitis J., Fury W., Miloscio L., Trejos J., Pangilinan J., Okamoto H., Cavino K., Na E., Papatheodorou A. (2017). Activin A More Prominently Regulates Muscle Mass in Primates than Does GDF8. Nat. Commun..

[36] Lee S.-J., Lehar A., Meir J.U., Koch C., Morgan A., Warren L.E., Rydzik R., Youngstrom D.W., Chandok H., George J. (2020). Targeting Myostatin/Activin A Protects against Skeletal Muscle and Bone Loss during Spaceflight. Proc. Natl. Acad. Sci. USA.

[37] Wetzlich B., Nyakundi B.B., Yang J. (2025). Therapeutic Applications and Challenges in Myostatin Inhibition for Enhanced Skeletal Muscle Mass and Functions. Mol. Cell. Biochem..

[38] Shuai Q., Tian J., Yang W., Huang S. (2026). Exosomes in osteoporosis: Regulatory mechanisms and clinical applications. Front Physiol..

[39] Mera P., Laue K., Ferron M., Confavreux C., Wei J., Galán-Díez M., Lacampagne A., Mitchell S.J., Mattison J.A., Chen Y. (2016). Osteocalcin Signaling in Myofibers Is Necessary and Sufficient for Optimum Adaptation to Exercise. Cell Metab..

[40] Dufresne S.S., Dumont N.A., Bouchard P., Lavergne É., Penninger J.M., Frenette J. (2015). Osteoprotegerin Protects against Muscular Dystrophy. Am. J. Pathol..

[41] Pin F., Jones A.J., Huot J.R., Narasimhan A., Zimmers T.A., Bonewald L.F., Bonetto A. (2022). RANKL Blockade Reduces Cachexia and Bone Loss Induced by Non-Metastatic Ovarian Cancer in Mice. J. Bone Miner. Res..

[42] China S.P., Pal S., Chattopadhyay S., Porwal K., Kushwaha S., Bhattacharyya S., Mittal M., Gurjar A.A., Barbhuyan T., Singh A.K. (2017). Globular Adiponectin Reverses Osteo-Sarcopenia and Altered Body Composition in Ovariectomized Rats. Bone.

[43] List E.O., Duran-Ortiz S., Kopchick J.J. (2020). Effects of Tissue-Specific GH Receptor Knockouts in Mice. Mol. Cell. Endocrinol..

[44] Laurent M.R., Dedeyne L., Dupont J., Mellaerts B., Dejaeger M., Gielen E. (2019). Age-Related Bone Loss and Sarcopenia in Men. Maturitas.

[45] Rajkomar A., Dean J., Kohane I. (2019). Machine Learning in Medicine. N. Engl. J. Med..

[46] Teo Z.L., Thirunavukarasu A.J., Elangovan K., Cheng H., Moova P., Soetikno B., Nielsen C., Pollreisz A., Ting D.S.J., Morris R.J.T. (2025). Generative Artificial Intelligence in Medicine. Nat. Med..

[47] Wändell P., Carlsson A.C., Swärd P., Eriksson J., Ärnlöv J., Rosenblad A., Wachtler C., Ruge T. (2025). A Machine Learning Tool for Predicting Newly Diagnosed Osteoporosis in Primary Healthcare in the Stockholm Region. Sci. Rep..

[48] Buccheri E., Dell’Aquila D., Russo M., Chiaramonte R., Musumeci G., Vecchio M. (2023). Can Artificial Intelligence Simplify the Screening of Muscle Mass Loss?. Heliyon.

[49] Lin Y.-T., Chu C.-Y., Hung K.-S., Lu C.-H., Bednarczyk E.M., Chen H.-Y. (2022). Can Machine Learning Predict Pharmacotherapy Outcomes? An Application Study in Osteoporosis. Comput. Methods Programs Biomed..

[50] Turimov Mustapoevich D., Kim W. (2023). Machine Learning Applications in Sarcopenia Detection and Management: A Comprehensive Survey. Healthcare.

[51] Goodman M.J., Ghate S.R., Mavros P., Sen S., Marcus R.L., Joy E., Brixner D.I. (2013). Development of a Practical Screening Tool to Predict Low Muscle Mass Using NHANES 1999-2004. J. Cachexia Sarcopenia Muscle.

[52] Chien K.-Y., Chen C.-N., Chen S.-C., Wang H.-H., Zhou W.-S., Chen L.-H. (2020). A Community-Based Approach to Lean Body Mass and Appendicular Skeletal Muscle Mass Prediction Using Body Circumferences in Community-Dwelling Elderly in Taiwan. Asia Pac. J. Clin. Nutr..

[53] Katano S., Yano T., Ohori K., Nagano N., Honma S., Shimomura K., Ishigo T., Watanabe A., Honma R., Fujito T. (2021). Novel Prediction Equation for Appendicular Skeletal Muscle Mass Estimation in Patients with Heart Failure: Potential Application in Daily Clinical Practice. Eur. J. Prev. Cardiol..

[54] Russo M. (2016). A Distributed Neuro-Genetic Programming Tool. Swarm Evol. Comput..

[55] Russo M. (2020). A Novel Technique to Self-Adapt Parameters in Parallel/Distributed Genetic Programming. Soft Comput..

[56] Zhang Y., Ma M., Huang X., Liu J., Tian C., Duan Z., Fu H., Huang L., Geng B. (2025). Machine Learning Is Changing Osteoporosis Detection: An Integrative Review. Osteoporos. Int..

[57] Bui H.M., Ha M.H., Pham H.G., Dao T.P., Nguyen T.-T.T., Nguyen M.L., Vuong N.T., Hoang X.H.T., Do L.T., Dao T.X. (2022). Predicting the Risk of Osteoporosis in Older Vietnamese Women Using Machine Learning Approaches. Sci. Rep..

[58] Li N., Ou J., He H., He J., Zhang L., Peng Z., Zhong J., Jiang N. (2024). Exploration of a Machine Learning Approach for Diagnosing Sarcopenia among Chinese Community-Dwelling Older Adults Using SEMG-Based Data. J. Neuroeng. Rehabil..

[59] Tu J.-B., Liao W.-J., Liu W.-C., Gao X.-H. (2024). Using Machine Learning Techniques to Predict the Risk of Osteoporosis Based on Nationwide Chronic Disease Data. Sci. Rep..

[60] Liu T., Luo Y.T., Pang P.C.I., Zhang H., Xiang A., Yang Q. (2026). The Role of Multimodal Generative AI in Older Adults’ Health Management: Systematic Scoping Review. JMIR AI.

[61] Vlietstra L., Hendrickx W., Waters D.L. (2018). Exercise Interventions in Healthy Older Adults with Sarcopenia: A Systematic Review and Meta-Analysis. Australas. J. Ageing.

[62] Ponzano M., Rodrigues I.B., Hosseini Z., Ashe M.C., Butt D.A., Chilibeck P.D., Stapleton J., Thabane L., Wark J.D., Giangregorio L.M. (2021). Progressive Resistance Training for Improving Health-Related Outcomes in People at Risk of Fracture: A Systematic Review and Meta-Analysis of Randomized Controlled Trials. Phys. Ther..

[63] Bauer J.M., Verlaan S., Bautmans I., Brandt K., Donini L.M., Maggio M., McMurdo M.E.T., Mets T., Seal C., Wijers S.L. (2015). Effects of a Vitamin D and Leucine-Enriched Whey Protein Nutritional Supplement on Measures of Sarcopenia in Older Adults, the PROVIDE Study: A Randomized, Double-Blind, Placebo-Controlled Trial. J. Am. Med. Dir. Assoc..

[64] Weaver C.M., Alexander D.D., Boushey C.J., Dawson-Hughes B., Lappe J.M., LeBoff M.S., Liu S., Looker A.C., Wallace T.C., Wang D.D. (2016). Calcium plus Vitamin D Supplementation and Risk of Fractures: An Updated Meta-Analysis from the National Osteoporosis Foundation. Osteoporos. Int..

[65] Fatima M., Brennan-Olsen S.L., Duque G. (2019). Therapeutic Approaches to Osteosarcopenia: Insights for the Clinician. Ther. Adv. Musculoskelet. Dis..

[66] Bauer J., Biolo G., Cederholm T., Cesari M., Cruz-Jentoft A.J., Morley J.E., Phillips S., Sieber C., Stehle P., Teta D. (2013). Evidence-Based Recommendations for Optimal Dietary Protein Intake in Older People: A Position Paper from the PROT-AGE Study Group. J. Am. Med. Dir. Assoc..

[67] Candow D.G., Forbes S.C., Chilibeck P.D., Cornish S.M., Antonio J., Kreider R.B. (2019). Effectiveness of Creatine Supplementation on Aging Muscle and Bone: Focus on Falls Prevention and Inflammation. J. Clin. Med..

[68] Mocini E., Cardinali L., Di Vincenzo O., Moretti A., Baldari C., Iolascon G., Migliaccio S. (2025). An Integrated Nutritional and Physical Activity Approach for Osteosarcopenia. Nutrients.

[69] Gonzalez Trotter D., Donahue S., Wynne C., Ali S., Parasoglou P., Boyapati A., Mohammadi K., Musser B.J., Meier P., Mastaitis J. (2025). GDF8 and Activin A Are the Key Negative Regulators of Muscle Mass in Postmenopausal Females: A Randomized Phase I Trial. Nat. Commun..

[70] Falsetti I., Palmini G., Donati S., Aurilia C., Iantomasi T., Brandi M.L. (2024). Irisin and Its Role in Postmenopausal Osteoporosis and Sarcopenia. Biomedicines.

[71] Colaianni G., Cinti S., Colucci S., Grano M. (2017). Irisin and Musculoskeletal Health. Ann. N. Y. Acad. Sci..

[72] Colaianni G., Cuscito C., Mongelli T., Pignataro P., Buccoliero C., Liu P., Lu P., Sartini L., Di Comite M., Mori G. (2015). The Myokine Irisin Increases Cortical Bone Mass. Proc. Natl. Acad. Sci. USA.

[73] Colaianni G., Mongelli T., Colucci S., Cinti S., Grano M. (2016). Crosstalk Between Muscle and Bone Via the Muscle-Myokine Irisin. Curr. Osteoporos. Rep..

[74] Nguyen N.H., Zhang P., Kadavan F.S.P., Xu Z., Nguyen T.T., Li W., Nguyen M.T., Nguyen C.K., Pham D.Q., Pham T.G.T. (2025). Multifunctional Hydroxyapatite Coated with Gallium Liquid Metal-Based Silver Nanoparticles for Infection Prevention and Bone Regeneration. Adv. Funct. Mater..

[75] Yousaf M.W., Nadeem A.H., Nadeem M.F., Qaisar R. (2026). The Role of Artificial Intelligence in Sarcopenia: Advances, Applications, and Future Directions. Comput. Biol. Chem..

[76] Fan S.-T., Lu M., Dong J.-L., Li Y.-L., Hao L.-N., Dong R.-C., Hou M.-D. (2025). Application of Artificial Intelligence in Osteoporosis: A Review. Front. Med..

